# Supplementary material for: Structural basis of GAIN domain autoproteolysis and cleavage-resistance in the adhesion G-protein coupled receptors
Source: Nat Commun. 2026 Apr 6;17:3259. doi: 10.1038/s41467-026-71225-1 (PMC13062082; doi:10.1038/s41467-026-71225-1)
Supplement: Supplementary file 1 — Supplementary Information [file 41467_2026_71225_MOESM1_ESM.pdf]

Supplementary Information

Structural basis of GAIN domain autoproteolysis and cleavage-resistance in the adhesion G-protein coupled receptors

Fabian Pohl, Florian Seufert, Yin Kwan Chung, Robin Schick, Björn Kieslich, Torsten Schöneberg, Tobias Langenhan, Peter W. Hildebrand, Norbert Sträter

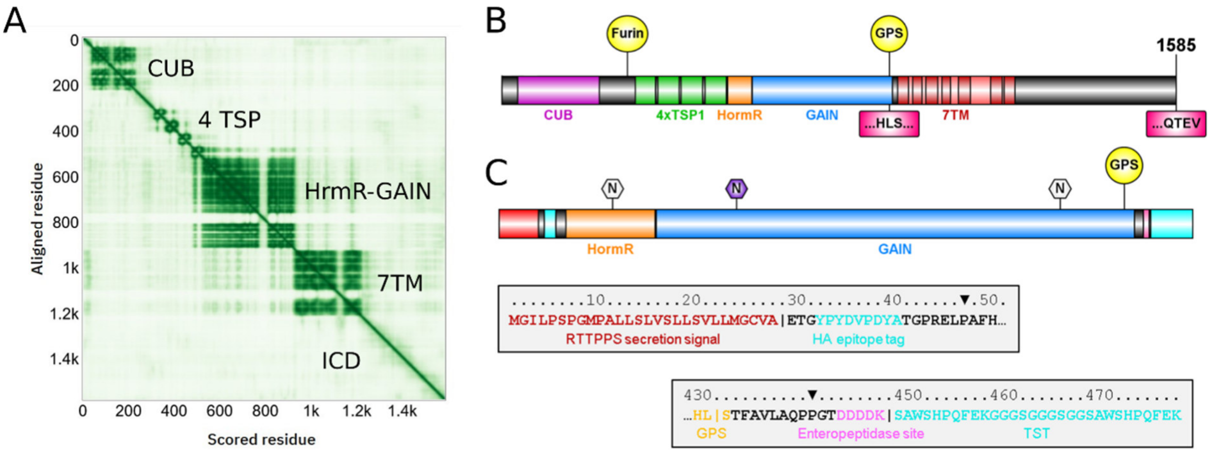

Supplementary Figure 1: Domain structure of ADGRB2. (A) Predicted Aligned Error (PAE, green for small errors, white for large errors) of the AlphaFold2 (AF2) prediction of the human B2 receptor. Low off-diagonal errors between domains are observed only for the HormR and GAIN domains, and to a lesser extent between the last thrombospondin (TSP) domain and the HormR-GAIN domains. This indicates that these regions are likely to have interactions and have a stable orientation relative to each other. In contrast, all other domains are predicted not to interact. The N-terminal domain of the AF2 prediction resembles most closely a CUB domain fold. Longer disordered (flexible) regions are predicted between the CUB and the first TSP domain and for the whole intracellular region. (B) Domains in the extracellular region of ADGRB2. (C) Construct *h*B2-HG used for crystal structure determination. The secretion signal is cleaved off before the mature protein is secreted into the medium. Possible N-glycosylation sites (N) were predicted by NetNGlyc 1.0. Those with a potential greater than 0.5 are shaded in purple. CUB – complement C1r/C1s, Uegf, Bmp1 (domain); GAIN – GPCR autoproteolysis inducing (domain); GPS – GPCR proteolytic site; HA – human influenza hemagglutinin; HormR – hormone receptor (domain); RTTPPS – receptor-type tyrosine-protein phosphatase S; TSP1 – thrombospondin-type 1 (repeat); TST – Twin-Strep-tag®; | denotes expected cleavage sites; ▼ denotes the insert's boundaries. The construct schemes in panels B and C were generated with IBS 2.0[1].

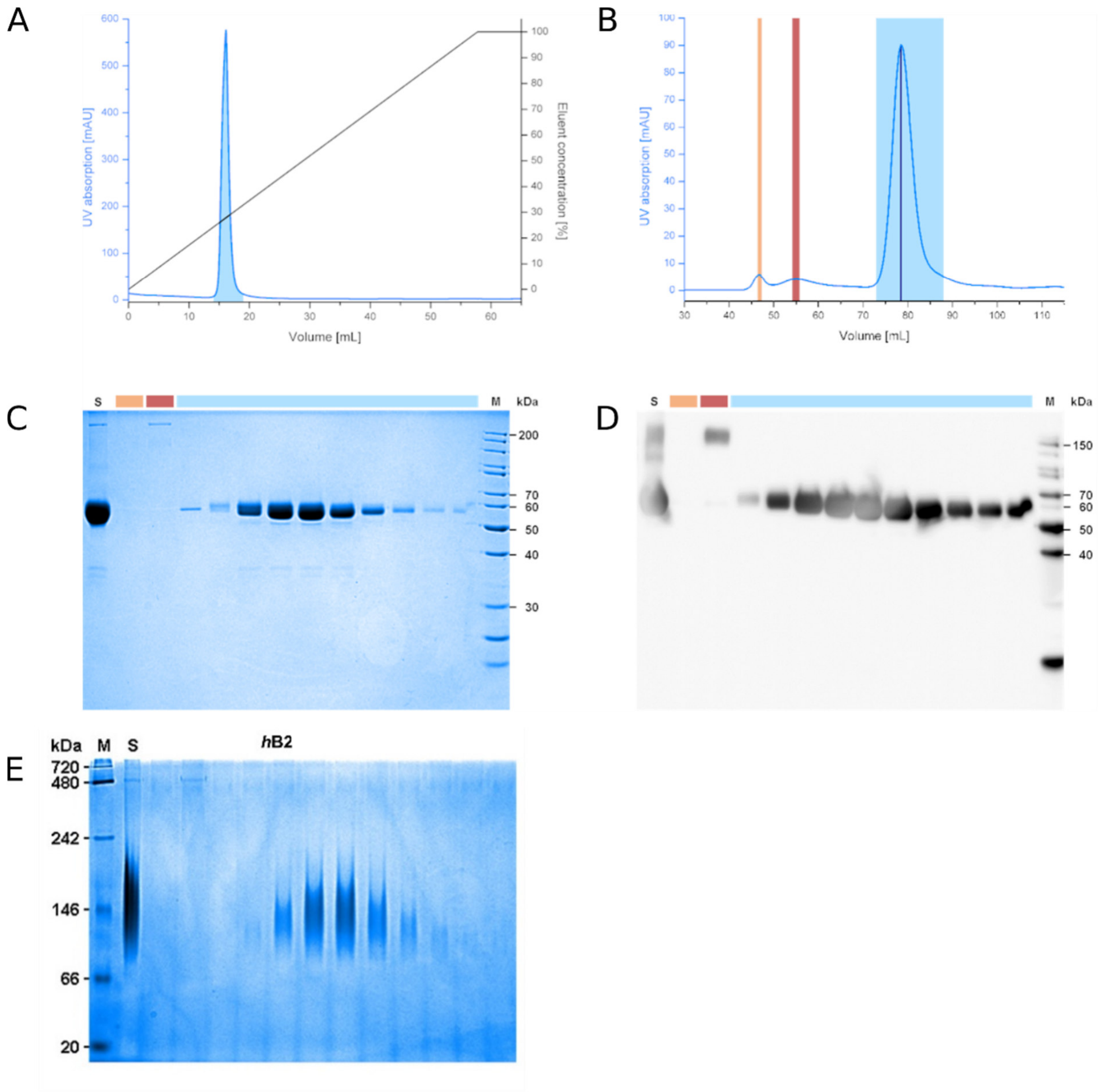

Supplementary Figure 2: Purification of *h*B2-HG. (A) Chromatograms of the affinity chromatography and size exclusion chromatography (SEC, B). (C) SDS-PAGE under nonreducing conditions and subsequent western blot detected via the C-terminal TST (D) of the SEC illustrates the purification progress. A small amount of cysteine-bridged oligomer (orange) was separated from the main peak (blue), which exhibited minor tailing in the SEC. Some degradation of the protein was visible as faint bands in the SDS-PAGE. (E) BN-PAGE analysis of the SEC fractions. M – marker; S – sample applied to the SEC. The theoretical masses for the *h*B2-HG construct are 55.4 kDa for a monomer and 110.8 kDa for a dimer. The apparent masses determined by SDS-PAGE and BN-PAGE are 52.3 kDa and 96.5 kDa, respectively. The calculated molecular weight is based on the mass of the amino acids and the putative N-glycosylation sites (1.217 kDa per high-mannose N-glycosylation site). O-glycosylation may contribute to the deviations between observed and calculated molecular masses.

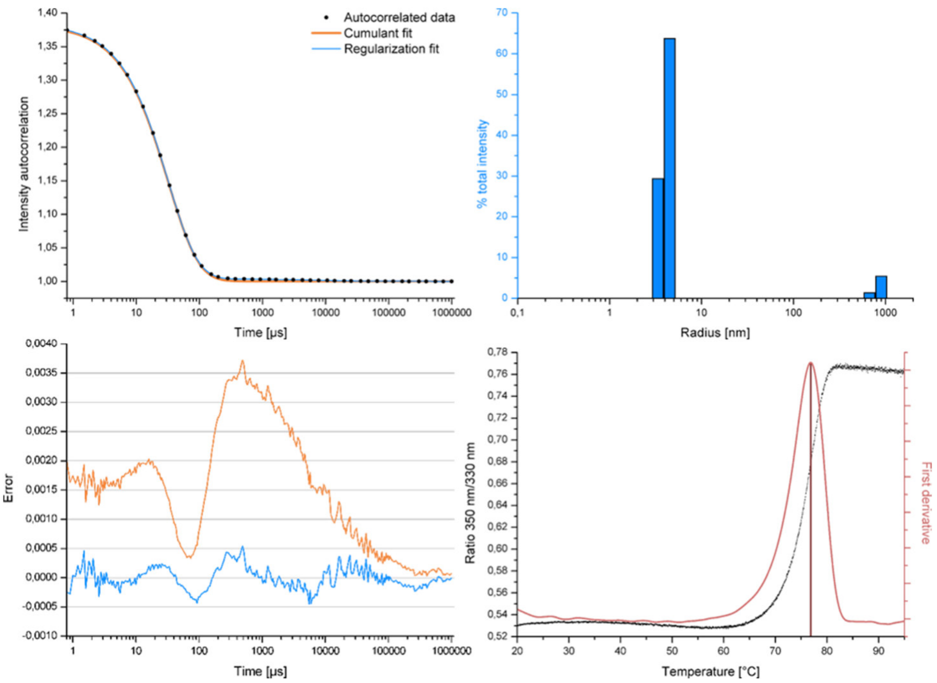

Supplementary Figure 3: Dynamic light scattering (DLS) and differential scanning fluorimetry (DSF) analysis of purified hB2-HG. The DLS autocorrelated data with cumulant and regularization fits is shown on the top left and the corresponding errors on the bottom left. The results of the regularization fit are visualized as a bar diagram on the top right. The DLS, measured at 10 mg/ml, showed a main species with a radius of ~4.1 nm (94 kDa for a spherical protein) and a polydispersity of 12.9 % with minor contamination by a larger, likely aggregated species. The DSF on the bottom right showed a sharp transition with a melting point of 76.6±0.08°C (n=3).

Supplementary Table 1: Crystallization of hB2-HG. Protein samples were used in the respective SEC buffers and their concentration is listed below. Crystals usually appeared within 48 h and kept slowly growing over time. Images shown here were taken after 34 days. Crystals appeared in ~12 out of ~700 tested conditions in the initial screening. The needle-like or plate-like crystals always grew from a common nucleation point as clusters.

Protein concentration	Crystallization condition	Crystals
*h*B2-HG<br>5 mg/ml	**M1B6**<br>100 mMsodium cacodylate, pH 6.5<br>200 mMmagnesium acetate<br>20 % (w/v) PEG 8,000	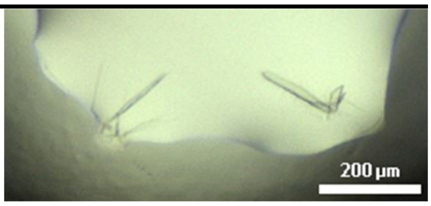
*h*B2-HG,<br>deglycosylated<br>5 mg/ml	**M1B6**<br>100 mMsodium cacodylate, pH 6.5<br>200 mMmagnesium acetate<br>20 % (w/v) PEG 8,000	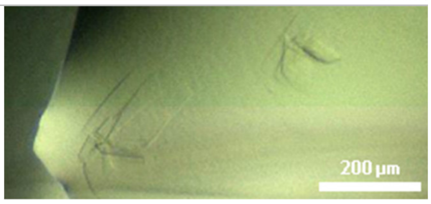
*h*B2-HG<br>5 mg/ml	**M4F3**<br>100 mMMES, pH 6.0<br>20 % (w/v) PEG 6,000	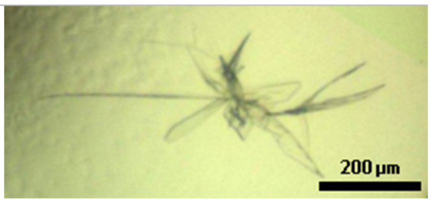

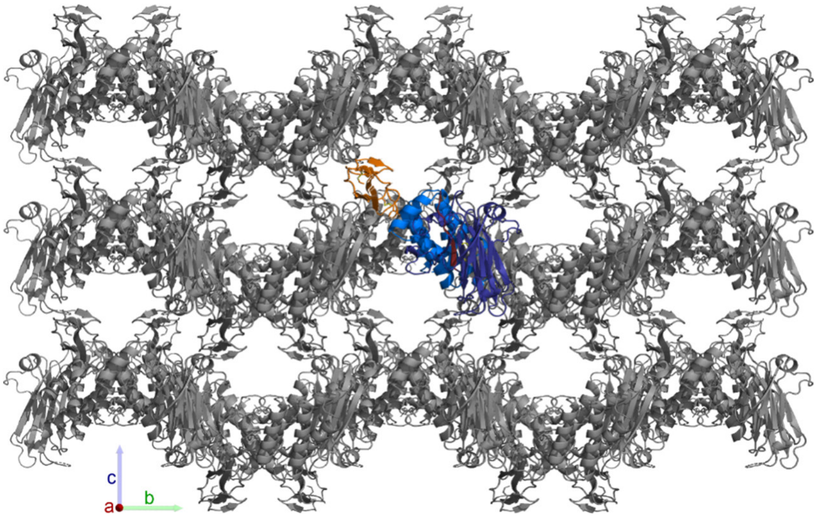

Supplementary Figure 4: Crystal packing of *h*B2-HG in space group $P2_12_12$. Crystals grew in layers with large channels between them. Crystal contacts between the layers are formed by the HormR domains. The axes shown on the bottom left align with the crystallographic unit cell. The weak contacts along the direction of unit cell axis **c** likely cause the platelike growth of the crystals (the **c**-axis located normal to the plates) and the anisotropic diffraction limits of 2.21 Å, 2.31 Å, and 3.02 Å along the **a***, **b***, and **c*** axes, respectively, observed for the crystal structure. The monomer is shown with the HormR domain in orange and the GAIN domain in blue.

Supplementary Table 2: Summary of crystallization, data collection and refinement

	***h*B2-HG (pdb ID 8oek)**
**Final crystal buffer**	100 mM sodium cacodylate, pH 6.7<br>200 mM magnesium acetate<br>18 % (w/v) PEG 8,000
**Data collection**	
Wavelength [Å]	1.77121
Resolution limits [Å]	63.34 - 2.22 (2.45 - 2.22)
Diffraction limits a*, b*, c* [Å]	2.21, 2.31, 3.02
Space group	$P2_12_12$
Unit cell a, b, c [Å]	84.86, 95.18, 49.46
Total reflections	164790 (6912)
Unique reflections	13145 (658)
Multiplicity	12.5 (10.5)
Completeness (spherical) [%]	63.8 (12.6)
Completeness (ellipsoidal [%]	89.1 (48.6)
Mean I/σ(I)	9.9 (1.3)
R_{meas}	0.223 (1.949)
$R_{pim.}$	0.062 (0.583)
CC½	0.997 (0.567)
Wilson B-factor [Å^2]	29.07
**Refinement**	
Resolution range [Å]	42.75 - 2.22 (2.30 - 2.22)
R_{work}	0.2325 (0.3803)
R_{free}	0.2869 (0.3499)

Number of non-hydrogen atoms	
Protein	2717
Heterogen	33
Solvent	18
B-factors [Å^2]	
Protein	35.39
Heterogen	49.20
Solvent	32.23
Ramachandran statistics [%]	
Favored	96.45
Allowed	3.25
Outliers	0.30
Root mean square deviation (RMSD)	
Bond lengths [Å]	0.002
Bond angles [°]	0.43

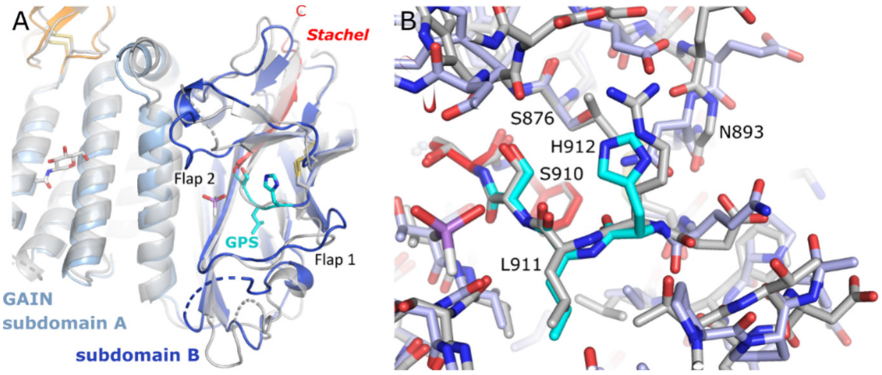

Supplementary Figure 5: Comparison of the GPCR proteolysis site (GPS) environment in the B2 and B3 GPCR autoproteolysis inducing (GAIN) domains. (A) Superposition of the crystal structures of the GAIN domains of *h*B2 (colors) and *h*B3 (grey, pdb id 4dlo). The two structures differ significantly in the fold of the flap regions around the GPS. (B) Closer view of the immediate GPS environment (B2 in colors, B3 in grey). Noteworthy differences are N893, which corresponds to the highly conserved glycine in B3 and in cleavage-competent GAIN domains, and S876, which corresponds to the phenylalanine or tyrosine residue forming an edge-π interaction with the histidine base (H911 in *h*B2) in the cleavage-competent receptors and is a leucine in *h*B3. Furthermore, the histidine base is replaced by an arginine residue in the GPS motif of *h*B3. Many other residues in the GPS environment also differ in sequence or conformation. The structures have been superimposed based on subdomain B of the GAIN domain.

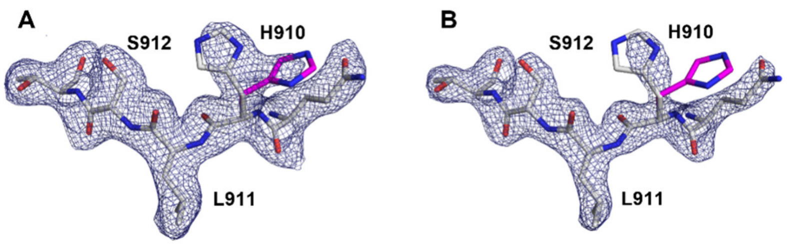

Supplementary Figure 6: Conformational flexibility of the H910 side chain in the *h*B2-HG crystal structure. The sidechain of H910 is present in two distinct orientations. The electron density around the GPCR proteolysis site (GPS) is shown at a contour level of 1.3 σ (A) and 0.6 σ (B). The two alternative rotamers of the H910 sidechain are shown in grey (refined occupancy 0.57) and magenta (0.43).

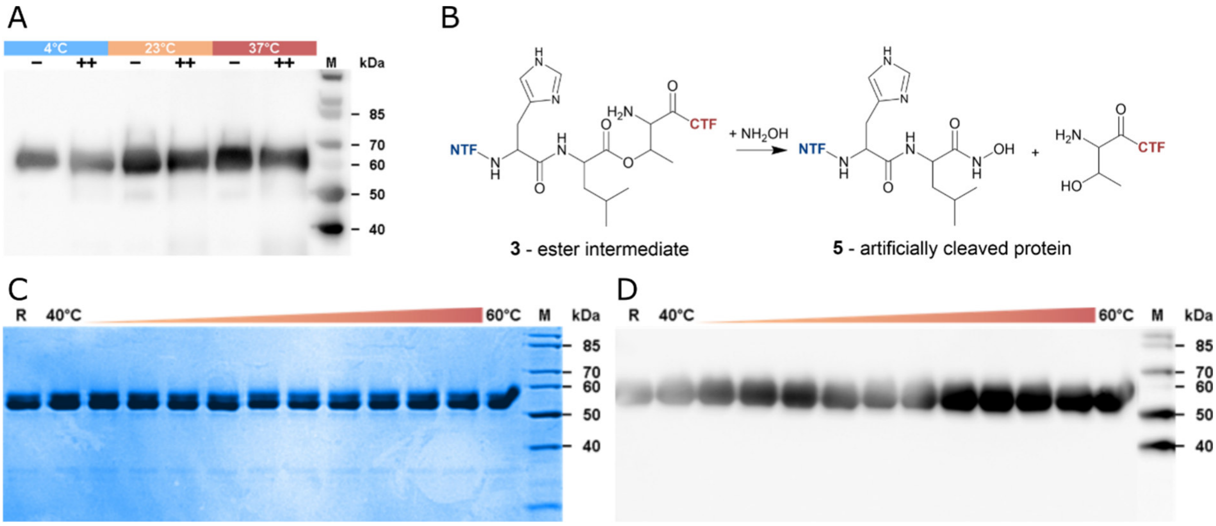

Supplementary Figure 7: The B2 GPCR autoproteolysis inducing (GAIN) domain is resistant to efficient cleavage at the GPCR proteolysis site (GPS) even under elevated temperatures or treatment with hydroxylamine. (A) Hydroxylamine assay of *h*B2-HG. – no hydroxylamine added; + 250 mM hydroxylamine; ++ 500 mM hydroxylamine; M – marker. The samples were incubated for overnight at the specified temperatures in a buffer containing 25 mM Tris pH 8.0 (determined at 4°C) and 150 mM NaCl prior to SDS-PAGE analysis. (B) Scheme of the hydrolysis reaction of an ester intermediate by hydroxylamine (C) Heat treatment of purified *h*B2-HG. The protein was incubated for 1 h at 40-60°C in a buffer containing 25 mM Tris pH 8.0 and 150 mM NaCl before the samples analyzed via SDS-PAGE under reducing conditions. (D) Western blot of the SDS-PAGE gel detected via the C-terminal TST. No shift in the apparent molecular weight or vanishing of bands in the western blot were observed. CTF/NTF C-/N-terminal fragment, M – Marker, R – Reference sample.

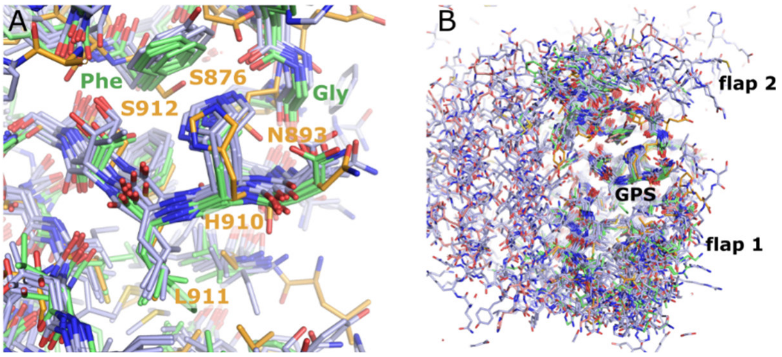

Supplementary Figure 8: Comparison of the GPCR proteolysis site (GPS) and its environment between the B2 GPCR autoproteolysis inducing (GAIN) domain and cleavage-competent receptors. (A) Comparison of the GPS and its environment between AlphaFold models of the following human receptors. Shown are cleavage-competent aGPCR for which structures of cleaved GAIN domains have been determined (green, L1, G1, G3 and G6) and for which cleavage has been reported mostly via gel-electrophoresis experiments: C2, D1, E2, E3, E5, F1, F3, F5, G2, G4, and L4 (blue). The crystallographic model of B2 is shown in orange. The histidine base of the GPS motif is oriented by an edge-π interaction with a phenylalanine (or tyrosine in E3) in cleavage-competent receptors whereas this residue is S912 in *h*B2. Furthermore, a highly conserved glycine residue is observed in autoproteolysis-active receptors at the position of N893 in *h*B2. (B) The wider GPS environment in these models differs tremendously due to the diversity and conformational flexibility of the flap regions and neighboring loops.

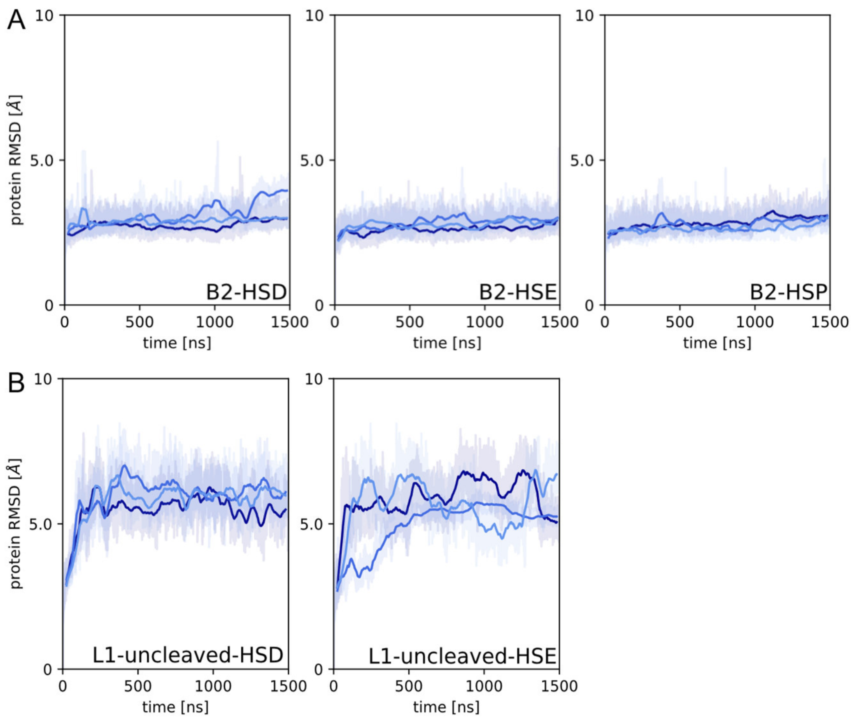

Supplementary Figure 9: Development of root mean square deviations (RMSD) to the starting structure during the time course of the MD simulations. (A) RMSD plots for the three replicas (dark blue: r1, medium blue: r2, bright blue: r3) of the three simulations (HSD, HSE and HSP) of the B2 GAIN domain. (B) RMSD plots for the HSD and HSE simulations of the L1 GAIN domain. The colored RMSD traces are averaged over a window of 20 frames, whereas the plots in grey indicate the RMSD fluctuations for each frame. HSD/HSE/HSP – Histidin side chain protonated at N_δ/N_ε/ N_δ and N_ε

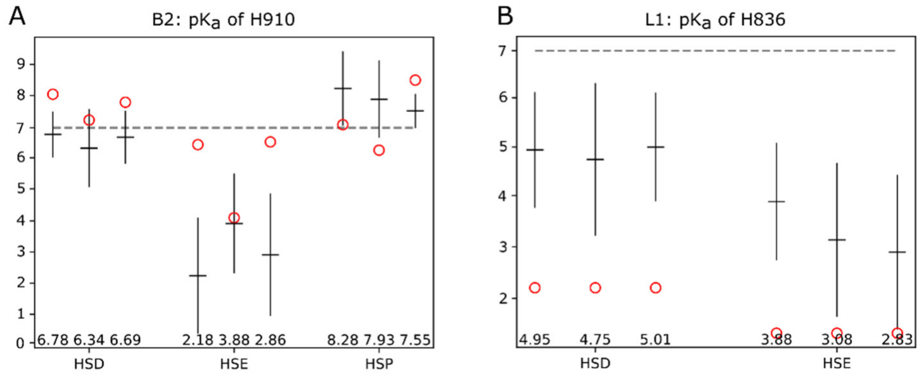

Supplementary Figure 10: Prediction of pK_a values for the catalytic histidine bases of the GPS during B2 and L1 MD simulations. (A) The pK_a value if H910 in B2 was predicted by solving the Poisson−Boltzmann equation for each frame of the three replica of the HSD (N_δ protonated), HSE (N_ε protonated) and HSP (both nitrogens protonated) protonation states. The average pK_a values are indicated by the horizontal line and specified numerically. The range of pK_a values is depicted by the vertical lines. Red circles mark the pK_a value of the starting structure after equilibration (B) pK_a values of H836 of the L1 simulations. As the HSE state results in MD conformations with the lowest pK_a values, the HSE protonation state is most stable at pH 7. HSD/HSE/HSP – Histidin side chain protonated at N_δ/N_ε/ N_δ and N_ε

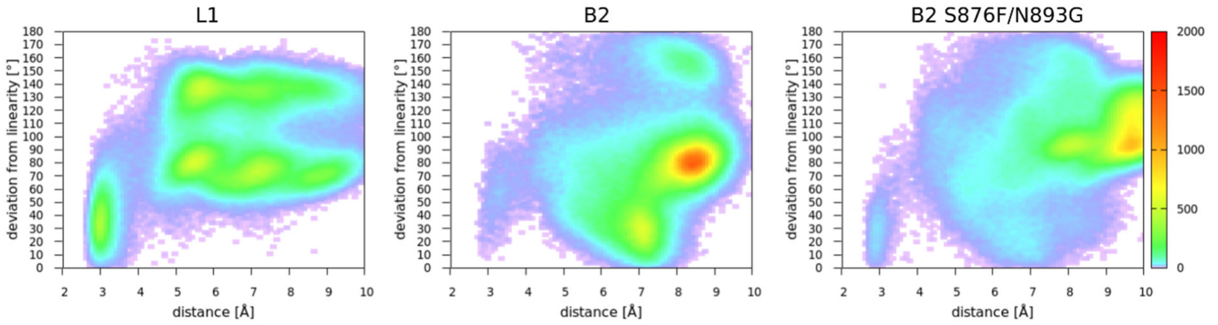

Supplementary Figure 11: Hydrogen bonding interactions between the His base and the Ser/Thr nucleophile in the MD simulation of L1, B2 and B2 S876F/N893G. The heatmaps show the frequency of occurrence of hydrogen bonding interactions between the N_δ atom of the histidine base and the O_γ atom of the Ser or Thr nucleophile by the distance between the two atoms and the deviation of the O_γ-H···N angle from linearity. A perfect hydrogen bond has a distance of ~2.8 Å and no deviation from linearity. A numerical evaluation of the number of MD frames, in which a hydrogen bond between the histidine base and the serine/threonine nucleophile is formed is given in Table 1 of the manuscript. The histidine base is in the HSE protonation state in these MD simulations.

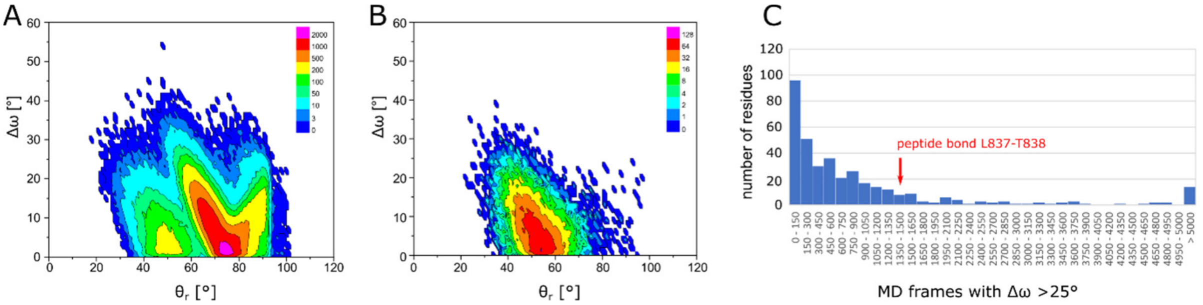

Supplementary Figure 12: Strain on the scissile peptide bond in the MD simulation of L1-HSE. (A) Heatmap of Δω (the deviation of the peptide bond from planarity) and Θ_r (the angular deviation from the perfect $\Theta_x = 90°$ and $\Theta_y = 90°$ orientation for attack of the alcohol nucleophile on the peptide bond). Δω and Θ_r was determined for all frames of the 3 replicas of the L1-HSE simulation. The scales indicate the color coding to visualize the number of frames for which a given pair of Δω and Θ_r (binned in 1° steps) is observed. The three maxima correspond to different rotamers of the T838 nucleophile. As only the p-rotamers of T838 result in reactive conformations of the GPS, only frames with this rotamer of T838 (criterium $\chi_1 = 59 \pm 30°$) are included in the heatmap in (B). With decreasing deviation from the ideal Θ angles for nucleophilic attack, the strain Δω of the scissile peptide increases. (C) Histogram showing how often residues of the HormR GAIN domains of L1 show strained peptide bonds (Δω > 25°) in replica 3 of the L1-HSE simulation. For example, around 85 residues have Δω angles larger than 25° in less than 150 of the 150706 frames of this simulation. Around 15 residues have strained peptide bonds in more than 5000 frames. For the scissile peptide bond L837-T838 Δω > 25° was observed in 1550-1650 frames. Thus, strained conformations occur for the scissile peptide bond more often than for most of the other residues (82.7 %).

Supplementary Table 3: Geometric parameters describing the orientation of the S/T alcohol nucleophile, the histidine base and the scissile peptide bond in the HSD (N_δ is protonated) MD simulations of the B2 and L1 GAIN domains. The values specify the number or percentage of frames in which the given condition is satisfied. These simulations were carried out with a neutral histidine base protonated at N_δ. Results from simulations with protonation at N_ε are shown in Table 1.

	$\Theta_x = 90° \pm 30°$ and $\Theta_y = 90° \pm 30°$	Hbond*	Hbond and $\Theta_{x/y} = 90° \pm 30°$	χ_1** His +60°, -60°, -180°	χ_1** Ser/Thr +60°, -60°, -180°
**B2 r1**	952	20	0	45.2%, 39.1%, 9.2%	7.2%, 9.8%, 74.3%
**B2 r2**	658	20	0	33.9%, 48.5%, 11.2%	7.7%, 8.5%, 73.9%
**B2 r3**	1054	18	0	47.9%, 36.7%, 8.6%	11.0%, 13.4%, 65.9%
**L1 r1**	3044	22	1	6.8%, 78.0%, 7.8%	42.0%, 1.1%, 24.7%
**L1 r2**	2109	9	0	10.6%, 66.4%, 15.6%	41.8%, 0.3%, 28.8%
**L1 r3**	27	0	0	5.1%, 72.4%, 10.2%	11.5%, 0.6%, 52.8%

*A favorable hydrogen bonding geometry was defined as a distance of 3.2 Å or less between the histidine nitrogen atom and the Ser/Thr O_γ atom and a deviation of less than 30° of the O_γ-H···N angle from linearity.

**The χ_1 torsion angle was assigned to the given value if it deviated by less than 30° from this value.

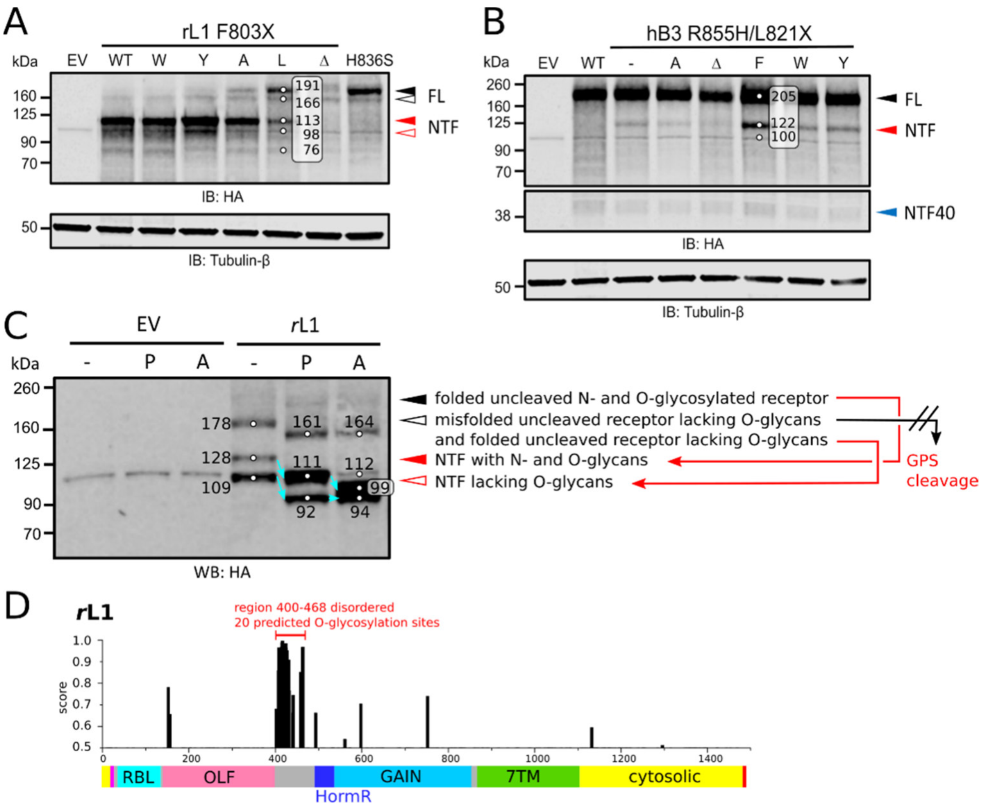

Supplementary Figure 13: Edge-π interaction between His[-2] base and surrounding residues is crucial for GPS cleavage of some receptors (further details and interpretation). (A,B) HEK293T cells were transiently transfected with constructs containing mutations causing either a disruption of edge-π interaction in *r*L1 (A), or a reintroduction of edge-π interaction in *h*B3 (B). GPS cleavage of the mutants was analyzed by western blots using 30 µl of cell lysates, targeted against the N-terminal HA tag of the receptors. Tubulin served as a loading control. Bands representing uncleaved and cleaved populations are indicated in black and red triangles, respectively. Fully and immaturely glycosylated populations of rat L1 are indicated in closed and open triangles, respectively. (C) *r*L1 is N- and O-glycosylated. HEK293T cells were transiently transfected with *r*L1. Lysates were treated with PNGase F ('P'), Deglycosylation mix II ('A') or without enzyme ('-'). Glycosylation states of receptors were examined by mobility shift of bands between treated and untreated samples (red dotted lines), detected against the N-terminal HA tag of the receptor. (D) Scheme of the *r*L1 construct used in these studies and positions and

probability (score, values higher than 0.5 are considered to indicate likely O-glycosylation sites) of O-glycosylation sites predicted by the algorithm of NetOGlyc-4.0 [2]. For the bands indicated by the small open circles, the apparent molecular mass has been estimated using the software GelAnalyzer 23.1.1 (www.gelanalyzer.com) to the values (in kDa) indicated next to the bands. 7TM - 7-transmembrane helix, EV – Empty vector, GAIN – GPCR autoproteolysis inducing, HA – Hemagglutinin, IB – Immunoblot, OLF – Olfactomedin-like, RBL - Rhamnose-Binding Lectin, WB – Western blot, WT – Wild type.

The theoretical molecular masses based on the construct sequences are 163.2 kDa (177.2kDa with 7 predicted N-glycosylation sites assuming an average mass of 2 kDa) for the full-length rat L1 construct used in this study, 92.8 kDa (106.8 kDa with 7 predicted N-glycans) for the *r*L1 NTF, 172.7 kDa (194.7 kDa with 11 predicted N-glycosylation sites) for the full-length *h*B3 construct and 98.0 kDa for the *h*B3 NTF (118 kDa with 10 predicted N-glycans). Higher masses likely result from O-glycosylation.

We interpret the observations in these WBs as follows. The upper bands in panel A (closed black triangle) correspond to properly folded protein with O- and N-glycosylation. This protein is principally prone to GPS autoproteolysis. The bands marked by the open black triangle correspond to protein without O-glycosylation (see below), that is stuck in the ER. The lack of O-glycosylation might result from misfolding, which would result in autocleavage deficiency independent of the GPS sequence. The Δ variant, which lacks a residue at position 836, cannot adopt a proper fold as the *Stachel* sequence is out of register and subdomain B cannot fold. Therefor only unfolded protein, but no cleaved protein is observed. The other GPS mutations result in proteins with correctly folded GAIN domains, that undergo GPS cleavage to different amounts. WT, F803W, and F803Y are fully cleaved at the GPS (except for the improperly folded protein), whereas F803A and F803L result in partial cleavage. H836S results in no GPS cleavage. Two bands are observed for the NTF, which result from differentially glycosylated proteins. The analysis in panel C demonstrates that the bands marked with an open black triangle correspond to N-glycosylated protein lacking O-glycosylation as treatment with PNGase F ("P"), which removes almost all N-glycans, results in the same shift as treatment with Deglycosylation mix II ("A"), a mixture of enzymes to remove all N-linked and simple O-linked glycans[1]. The bands marked with a closed red triangle contain N- and O-glycosylation, whereas the bands marked with an open triangle contain only N-glycosylated protein. The thickness of the lower band(s) in experiment rL1-A in panel C and its height relative to the lower band of rL1-P indicates that some of the O-glycosylation of the bands marked with the open red triangle has not been removed. The mutations of residue F803 in *r*L1 (panel A) which reduce GPS cleavage activity also reduce the fraction of cleaved protein lacking O-glycosylation. The reason for this behavior is currently not clear.

The deglycosylation enzymes used in experiments "P" and "A" differ also in the activity to remove N-glycans. Whereas PNGase F ("P") cannot remove N-glycans that have an α1-3-linkage to a fucose at the first GlcNAc residue, deglycosylation mix II ("A") would remove such glycans. The *r*L1 construct used for this study contains nine predicted N-glycosylation sites (score > 0.5 using prediction server NetNGlyc-1.0[3]). Assuming an average mass of ~2 kDa per predicted glycosylation site in HEK293T cell expression, this may result in ~18 kDa molecular mass resulting from N-glycosylation. Since deglycosylation with PNGase F already results in a mass shift of ~17 kDa and application of the Deglycosylation mix II in a mass shift of ~34 kDa, it appears unlikely that the additional mass shift observed for the deglycosylation mix has large contributions from α1-3-fucosylated GlcNAc residues at the first glycan position. The mass shift is mostly due to the removal of O-glycans.

[1]https://international.neb.com/products/p6044-protein-deglycosylation-mix-ii#Product%20Information

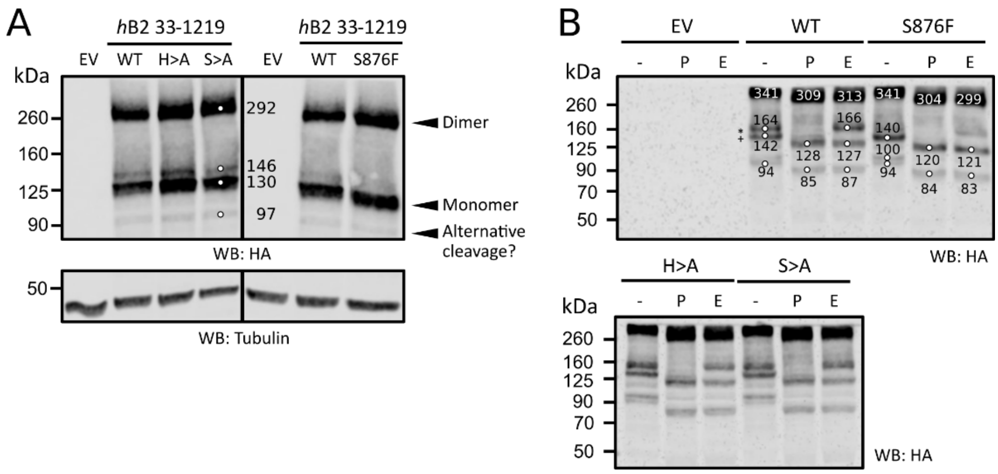

Supplementary Figure 14: Autoproteolysis is not observed for the full-length *h*B2. HEK293T cells were transiently transfected with indicated mutated constructs of *h*B2 residues 33-1219. (A) Western blotting of an SDS-PAGE showed expression of *h*B2 and respective mutants targeted against the N-terminal HA tag of the receptor. 30 µl of lysate samples were used for analysis. Tubulin served as loading control. For the bands indicated by the small open circles, the apparent molecular mass has been estimated using the software GelAnalyzer 19.1 (www.gelanalyzer.com) to the values (in kDa) indicated next to the bands. (B) Western blotting of SDS-PAGE of *h*B2 lysates treated with PNGase F ("P"), Endo H ("E"), or without any enzyme treatment (-). Endo H-resistant and sensitive bands of monomeric *h*B2 indicated by "*" and "+", respectively. The ectodomain of B2 contains five probable and three further N-glycosylation sites of lower probability. The calculated molecular masses of the construct used are 137.6 kDa (157.6 kDa with 8 N-linked complex glycans of 2.5 kDa) for full-length *h*B2, 32.8 kDa (40.3 kDa with 3 N-glycans) for the N-terminal fragment up to the furin cleavage site, 100.4 kDa (120.4 kDa with 8 N-glycans) for the NTF. We interpret the bands observed at ~130 kDa and ~290 kDa as uncleaved full-length receptor migrating as monomer and dimer. Membrane proteins can display significant anomalies in migration behavior in SDS-PAGE[4]. O-glycosylation likely contributes to further deviations from the expected molecular mass.

The band marked as "Alternative cleavage?" is observed in all samples at an apparent molecular mass of ~90 kDa. This band is also observed in the GPS mutants H910A and S912A, which are highly unlikely to possess autoproteolytic activity. These fragments may result in cleavage of the long, disordered loop 770-810, which would generate a fragment of calculated mass of 87.2 kDa (104.7 kDa for 7 N-glycans) for cleavage at position 790. A similar fragment (named Vstat-40) has been observed for *h*B1[5]. PNGase F removes the complete glycan chain for all types of N-linked glycosylation (high mannose, hybrid, bi-, tri-, and tetra-antennary), unless the innermost GlcNAc residue is α1-3-linked to a fucose. Endo H removes only high mannose and some hybrid types of N-linked carbohydrates by cleavage between the first and second GlcNAc residues. The two bands observed at apparent molecular masses of 164 kDa and 142 kDa for the deglycosylation experiment in panel (B) differ therefor in N-glycosylation. Band "*" contains additional complex glycans that are not removed by EndoH. Band "+" contains only glycans that can be removed by Endo H. It is interesting to note that predominantly only two distinct bands are observed. These differ in the maturation state of several glycans such that all are present as complex glycans in "*" and are missing or present in a state that the can be processed by Endo H in "+". The lower immaturely glycosylated band at 142 kDa may indicate misfolded protein as a result of overexpression.

EV – Empty vector, HA – Hemagglutinin, WB – Western blot, WT – Wild type

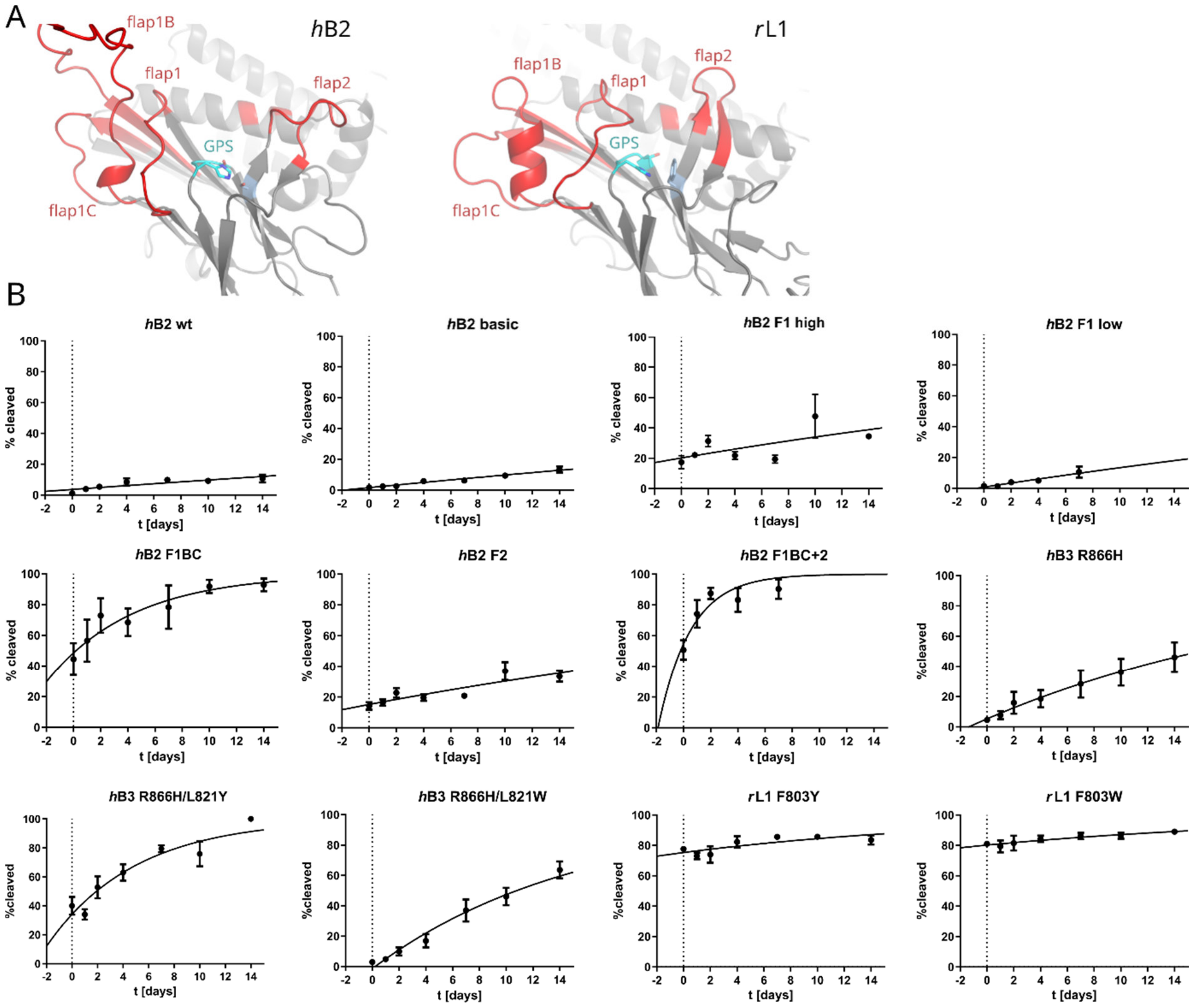

Supplementary Figure 15: Analysis of GPCR proteolysis site (GPS) autocleavage and structure of the investigated variants of *h*B2, *h*B3 and *r*L1. (A) Cartoon representations of the *h*B2 and *r*L1 ectodomains. The regions of flap exchange are colored red. (B) Analysis of the kinetics of GPS autocleavage for selected variants of the *h*B2, *h*B3 and *r*L1 ectodomains. The flap exchange chimeras have been referred to by the following abbreviations. F1: flap 1, F1BC: flap 1 + 1B + 1C, F2: flap 2, F1+2: flap 1 + 2, F1BC+2: flap 1 + 1B + 1C + 2. Refer to the supplementary data fasta file for the sequences of the variants. "*h*B2 F1 high" and "*h*B2 F1 low" are two experiments using the same variant and conditions, but the initial amount of cleavage differs so much that the data have been evaluated independently. "*h*B2 basic" is the S876F/D890S/E892Q/N893G variant. For *h*B3 R866H and *h*B3 R886H/L821W we obtained 3, for *h*B2 basic, *h*B2 F1, *h*B2 F2, *h*B2 F1BC, *h*B3 wt, *r*L1 F803Y and *r*L1 F803W we obtained two and for *h*B2 wt, *h*B2 DM, *h*B2 F1BC+2 and *h*B3 R866H/L821Y one biological replica (independent expression and purification). Two technical replica (quantifications via western blot) were measured for each independent expression and purification. Data are presented as mean values +/- SD.

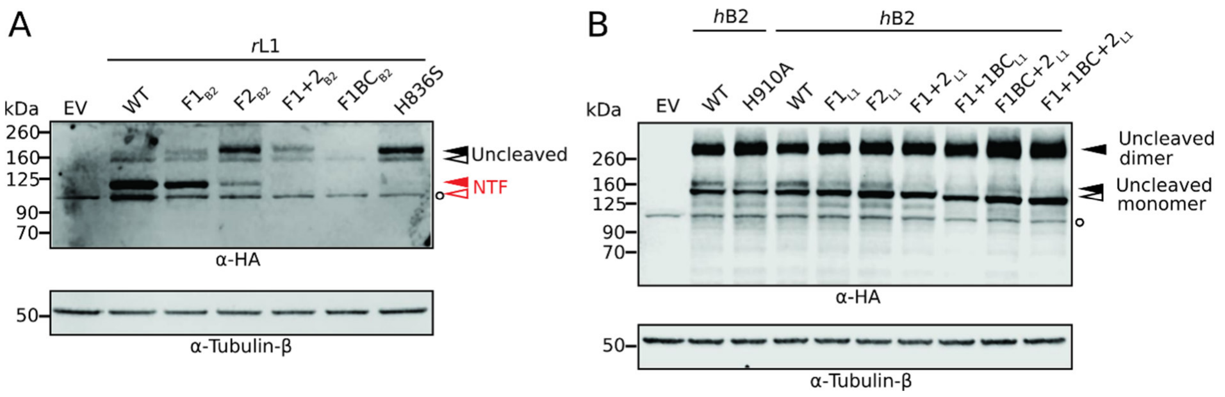

Supplementary Figure 16: GPS autoproteolysis of flap exchange variants of full-length *r*L1 and *h*B2 receptors. (A) Western blot analysis of the full-length *r*L1 receptor after expression in HEK293T cells. "EV" denotes the empty expression vector, "WT" the wild-type receptor, F1 the exchange of flap 1 against that of the *h*B2 receptor (see Fig. S15A), F2 the flap 2 exchange, F1+2 the exchange of flaps 1 and 2, and F1BC the exchange of flaps 1, 1B and 1C. The variations are the same as those of the corresponding ectodomain constructs (Fig. 8, Fig. S15). The filled and open triangles mark differently glycosylated protein as described and analyzed in Fig. S13. (B) Western blot analysis of flap exchange mutants and controls of the full-length *h*B2 receptor. As described and analyzed in Figure S13, the dominant lower band of the monomeric protein marked with an open triangle likely corresponds to immaturely glycosylated protein. We suspect that this protein is misfolded as a result of overexpression and therefore lacks the autoproteolytic activity observed for the secreted and purified *h*B2-HG constructs. EV – Empty vector, HA – Hemagglutinin, WT – Wild type

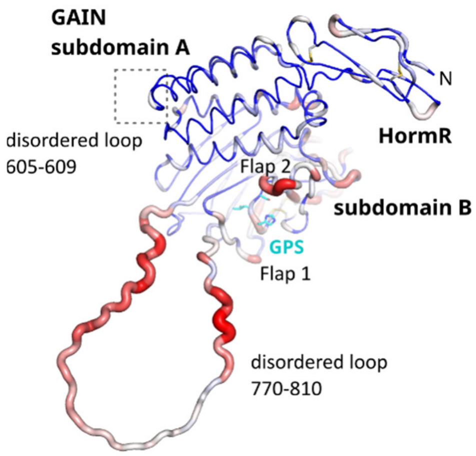

Supplementary Figure 17: Conservation of the long flexible loop 770-810 of B2. Sequences of the HormR and GAIN domains of 148 mammalian species were aligned and the Shannon entropy was calculated for each residue, which quantifies the variability of the sequence positions. Residues of high conservation are shown as blue thin tube regions and residues of high sequence variation as red thick tube regions. This figure includes the loops of residues 605-609 and 770-810, which are disordered in the crystal structure. The loops are modeled in arbitrary conformations only for visualization of the residue conservation. GAIN – GPCR autoproeolysis inducing, HormR – Hormone receptor.

Supplementary Table 4: Comparison of the GPS of GAIN domains to other proteins with *cis*-autoprocessing activity

protein	sequence	base	oxoanion hole	protonation amide NH	strain at or near scissile peptide bond	inactivation, pdb id	ref
NtN hydrolases: glycosylasparaginase	^{151}D↓TI	D151	T170-OH	D151 suggested*[1]	on scissile peptide bond and/or penultimate peptide bond	T152C, 3ljq	[6,7]
Pantetheine hydrolase ThnT	^{281}N↓TT	no	N217-$N_{\delta}H_2$, F144-NH	no	two conformations of main chain in T282C variant. Strained φ,ψ-angles of C282 in state A. Strained ω angle in state B.	T282C, 3s3u	[8]
Nup98	^{862}HF↓S	H862	K791-NH_3^+, N799-NH_2	H862?, not discussed in ref.[9]	Strained scissile *cis*-peptide bond	S864A, 2q5x	[9]
SEA domains: *h*Muc1 SEA domain	^{1088}G↓SVVV	no	no	no	Substantial strain of 7 kcal/mol, scissile bond also strained	modeled	[10,11]
Inteins: GyrA	^{0}T↓CI*[2]	no	T72-OH (N74-NH_2)	H75	Scissile bond is *cis*-peptide, no strain, but *cis*-peptide has ~5 kcal/mol higher energy	^{0}A↓SI, 1am2	[12]
Hedgehog proteins: Drosophila hedgehog	^{256}HG↓CF	no	T326-OH	H329	no uncleaved structure available	cleaved: 1at0	[13]
Pyruvoyl enzymes: S-Adenosylmethionine decarboxylase	^{66}SE↓SS	no*[3]	no, C82-SH?	wat2 and H243	no	S68A, 1msv	[14]

*[1]D151 was suggested to protonate the leaving amide nitrogen, but it is unclear if a direct proton transfer is possible; *[2]T is the last residue of the extein; *[3]the authors suggest that the proton of the serine nucleophile is directly transferred to the carbonyl oxygen of the scissile peptide bond.

Supplementary Table 5: Antibodies and reagents for western blotting

Protein purification and characterization assays

- Strep-Tactin HRP conjugate (Life Sciences; 2-1502-001) 1:75000
- HA-antibody HRP (Miltenyi Biotech; 130-091-972) 1:5000
- Anti-His6 peroxidase (Sigma-Aldrich; 11965085001 1:1000

Full-length autoproteolysis assays

- HA-Tag (C29F4) Rabbit mAb #3724 (Cell Signaling Technology) 1:1000
- Tubulin-beta (E7) mouse mAb (DSHB) 1:5000
- IRDye(R) 680RD Goat anti-Rabbit IgG secondary antibody (#926-68071) (Licor) 1:15000
- IRDye(R) 800CW Goat anti-Mouse IgG secondary antibody (#926-32210) (Licor) 1:15000

References

1. Xie, Y. *et al.* IBS 2.0: an upgraded illustrator for the visualization of biological sequences. *Nucleic acids research* **50,** W420-W426; 10.1093/nar/gkac373 (2022).
2. Steentoft, C. *et al.* Precision mapping of the human O-GalNAc glycoproteome through SimpleCell technology. *The EMBO journal* **32,** 1478–1488; 10.1038/emboj.2013.79 (2013).
3. Gupta, R. & Brunak, S. Prediction of glycosylation across the human proteome and the correlation to protein function. *Pacific Symposium on Biocomputing. Pacific Symposium on Biocomputing,* 310–322 (2002).
4. Rath, A., Glibowicka, M., Nadeau, V. G., Chen, G. & Deber, C. M. Detergent binding explains anomalous SDS-PAGE migration of membrane proteins. *Proceedings of the National Academy of Sciences of the United States of America* **106,** 1760–1765; 10.1073/pnas.0813167106 (2009).
5. Cork, S. M. *et al.* A proprotein convertase/MMP-14 proteolytic cascade releases a novel 40 kDa vasculostatin from tumor suppressor BAI1. *Oncogene* **31,** 5144–5152; 10.1038/onc.2012.1 (2012).
6. Xu, Q., Buckley, D., Guan, C. & Guo, H. C. Structural insights into the mechanism of intramolecular proteolysis. *Cell* **98,** 651–661; 10.1016/s0092-8674(00)80052-5 (1999).
7. Wang, Y. & Guo, H.-C. Crystallographic snapshot of glycosylasparaginase precursor poised for autoprocessing. *Journal of molecular biology* **403,** 120–130; 10.1016/j.jmb.2010.08.038 (2010).
8. Buller, A. R., Freeman, M. F., Wright, N. T., Schildbach, J. F. & Townsend, C. A. Insights into cis-autoproteolysis reveal a reactive state formed through conformational rearrangement. *Proceedings of the National Academy of Sciences of the United States of America* **109,** 2308–2313; 10.1073/pnas.1113633109 (2012).
9. Sun, Y. & Guo, H.-C. Structural constraints on autoprocessing of the human nucleoporin Nup98. *Protein science : a publication of the Protein Society* **17,** 494–505; 10.1110/ps.073311808 (2008).
10. Johansson, D. G. A., Macao, B., Sandberg, A. & Härd, T. SEA domain autoproteolysis accelerated by conformational strain: mechanistic aspects. *Journal of molecular biology* **377,** 1130–1143; 10.1016/j.jmb.2008.01.050 (2008).
11. Johansson, D. G. A. *et al.* Protein autoproteolysis: conformational strain linked to the rate of peptide cleavage by the pH dependence of the N -- O acyl shift reaction. *Journal of the American Chemical Society* **131,** 9475–9477; 10.1021/ja9010817 (2009).
12. Klabunde, T., Sharma, S., Telenti, A., Jacobs, W. R. & Sacchettini, J. C. Crystal structure of GyrA intein from Mycobacterium xenopi reveals structural basis of protein splicing. *Nature structural biology* **5,** 31–36; 10.1038/nsb0198-31 (1998).
13. Hall, T. M. *et al.* Crystal structure of a Hedgehog autoprocessing domain: homology between Hedgehog and self-splicing proteins. *Cell* **91,** 85–97; 10.1016/s0092-8674(01)80011-8 (1997).
14. Tolbert, W. D. *et al.* Mechanism of human S-adenosylmethionine decarboxylase proenzyme processing as revealed by the structure of the S68A mutant. *Biochemistry* **42,** 2386–2395; 10.1021/bi0268854 (2003).
