# Supplementary material for: Structural basis of GAIN domain autoproteolysis and cleavage-resistance in the adhesion G-protein coupled receptors
Source: Nat Commun. 2026 Apr 6;17:3259. doi: 10.1038/s41467-026-71225-1 (PMC13062082; doi:10.1038/s41467-026-71225-1)
Supplement: Supplementary file 2 — Description of Additional Supplementary Files [file 41467_2026_71225_MOESM2_ESM.pdf]

Description of Additional Supplementary Files

File name: Supplementary Data 1
Description: List of primers and plasmids used in this study
