# Supplementary material for: Structural basis of GAIN domain autoproteolysis and cleavage-resistance in the adhesion G-protein coupled receptors
Source: Nat Commun. 2026 Apr 6;17:3259. doi: 10.1038/s41467-026-71225-1 (PMC13062082; doi:10.1038/s41467-026-71225-1)
Supplement: Supplementary file 3 — Supplementary Data 1 [file 41467_2026_71225_MOESM3_ESM.pdf]

List of primers used in this study

Primer ID	Sequence (5'→3')
F803W_for	TGCAGCTGGTGGAACTACAGCGAGAG
F803W_rev	GTTCCACCAGCTGCAGTTGGCGTTG
F803Y_for	CAGCTACTGGAACTACAGCGAG
F803Y_rev	TCCAGTAGCTGCAGTTGGCGTTG
F803A_for	CAGCGCCTGGAACTACAGCGAG
F803A_rev	TCCAGGCGCTGCAGTTGGCGTTG
F803L_for	CAGCTTGTGGAACTACAGCGAG
F803L_rev	TCCACAAGCTGCAGTTGGCGTTG
H836S_for	CAGCAGCCTGACCAATTTCGCCGTG
H836S_rev	TCAGGCTGCTGCAGGCACAGGTG
rL1_HG_AgeI_for	AATTAACCGGTCCTGCTCCTAGCACAAG
rL1_HG_KpnI_rev	TTTAGGGTACCGTAGATCTCTCTGTGGGCCATC
R855H_for	TGCGATCACCTGAGCACCTTCGCCATC
R855H_rev	GCTCAGGTGATCGCACAGGCACTTG
L821W_for	TGCGTGTGGTGGGACGACAGCAAG
L821W_rev	GTCCCACCACACGCAGTAAGGATTCAG
L821Y_for	TGCGTGTATTGGGACGACAGCAAGAC
L821Y_rev	GTCCCAATACACGCAGTAAGGATTCAGTG

L821F_for	TGCGTGTTTTGGGACGACAGCAAG
L821F_rev	GTCCCAAAACACGCAGTAAGGATTCAG
L821A_for	TGCGTGGCGTGGGACGACAGCAAG
L821A_rev	GTCCCACGCCACGCAGTAAGGATTCAG
hB3_HG_AgeI_for	TCATTACCGGTGAAGTGCGGAGATGTAG
hB3_HG_KpnI_rev	TATTAGGTACCCTCTCTTGGCTGCTGTG
Primers used for the full-length constructs	
ac_1F	TAATACGACTCACTATAGGG
ac_21R	GCTCTAGCATTTAGGTGACAC
ac_102F	AACTGCTCCTGGAACTACTCA
ac_103R	TGAGTAGTTCCAGGAGCAGTT
ac_104F	AACTGCTCCGCCTGGAACTACTCA
ac_105R	TGAGTAGTTCCAGGCGGAGCAGTT
ac_106F	AACTGCTCCTTATGGAACTACTCA
ac_107R	TGAGTAGTTCCATAAGGAGCAGTT
ac_110F	AACTGCTCCTGGTGGAACTAC
ac_111R	GTAGTTCCACCAGGAGCAGTT
ac_128F	CTTCAATGCAAACTGCTCCTACTGGAACTACTCAGAGCGC
ac_129R	GCGCTCTGAGTAGTTCCAGTAGGAGCAGTTTGCATTGAAG
ac_168F	CACCTGGCTACCTTTGCCGTGCTG

ac_169R	AAAGGTAGCCAGGTGCTGACACTGGCAT
ac_170F	CAGTGTCAGGCCCTGTCTACC
ac_171R	GGTAGACAGGGCCTGACACTG
ac_172F	CACTGCGCCTTCTGGGATTAC
ac_173R	GTAATCCCAGAAGGCGCAGTG
B2_528-X_SacI	ATTATGAGCTCCCTGCCTTCCACGAGATGTG
B2_y-921_KpnI	ATATAGGTACCAGGAGGTTGAGCCAGCACG

List of plasmids used in this study

Site-directed mutagenesis was performed using *Phusion* High-Fidelity DNA polymerase (ThermoFisher). PCR amplifications of fragments was made using *Phusion* High-Fidelity DNA polymerase (ThermoFisher). Ligations were done by Quick Ligase (NEB). Reactions were designed following manufacturers' instructions. The numbering of the mutations follows that of the canonical sequence of the receptor under the Uniprot entry. All plasmids were confirmed by sequencing before downstream applications. The complete amino acid sequences of the flap exchange variants are specified in a document in the Zenodo deposition DOI:10.5281/zenodo.1549016.

Vectors for the expression of the full-length constructs

Construct	ID	Descriptions	Tags	Construction method	Primers used
$L1^{WT}$	pSA132	Overexpression of rat L1 in pcDNA3.1 (Uniprot: O88917-2)	HA/Flag	-	-
$L1^{H836S}$	pSA163	GPS-mutated, cleavage-deficient (H>S) L1	HA/Flag	-	-

$L1^{F803W}$	pAC71	Mutation on the aromatic counterpart (F>W) of the edge-π interaction of L1	HA/Flag	Ligation of two fragments of 2.4kb and 2kb from pSA132 with 5.3kb fragment of pSA25 (HindIII/XbaI)	2.4kb fragment: ac_1F and ac_111R;<br>2kb fragment: ac_110F and ac_21R
$L1^{F803Y}$	pAC95	Mutation on the aromatic counterpart (F>Y) of the edge-π interaction of L1	HA/Flag	Ligation of two fragments of 2.4kb and 2kb from pSA132 with 5.3kb fragment of pSA25 (HindIII/XbaI)	2.4kb fragment: ac_1F and ac_129R;<br>2kb fragment: ac_128F and ac_21R
$L1^{F803A}$	pAC68	Mutation on the aromatic counterpart (F>A) of the edge-π interaction of L1	HA/Flag	Ligation of two fragments of 2.4kb and 2kb from pSA132 with 5.3kb fragment of pSA25 (HindIII/XbaI)	2.4kb fragment: ac_1F and ac_105R;<br>2kb fragment: ac_104F and ac_21R
$L1^{F803L}$	pAC60	Mutation on the aromatic counterpart (F>L) of the edge-π interaction of L1	HA/Flag	Ligation of two fragments of 2.4kb and 2kb from pSA132 with 5.3kb fragment of pSA25 (HindIII/XbaI)	2.4kb fragment: ac_1F and ac_107R;<br>2kb fragment: ac_106F and ac_21R
$L1^{F803\Delta}$	pAC70	Deletion on the aromatic counterpart (F803) of the edge-π interaction of L1	HA/Flag	Site-directed mutagenesis with pSA132 as template	ac_102F and ac_103R
$B2(aa33\text{-}1219)^{WT}$	pAC141	Overexpression of human B2 in pcDNA3.1, spanning 33rd to 1219th residues (Uniprot: O60241-1)	HA	Ligation of a fragment of 3.8kb from pAC139 (B2aa33-1219 in pHLSec, gift from Norbert Sträter) with 5.3kb fragment of pSA25 (EcoRI/XhoI)	-
$B2(aa33\text{-}1219)^{H910A}$	pAC146	GPS-mutated (H>A) B2	HA	Ligation of two fragments of 1.2kb and 2.8kb from pAC141 with 5.3kb fragment of pSA25 (EcoRI/XhoI)	1.2kb fragment: ac_170F and ac_21R;<br>2.8kb fragment: ac_1F and ac_171R

B2(aa33-1219)^{S912A}	pAC147	GPS-mutated (S>A) B2	HA	Ligation of two fragments of 1.2kb and 2.8kb from pAC141 with 5.3kb fragment of pSA25 (EcoRI/XhoI)	1.2kb fragment: ac_168F and ac_21R;<br>2.8kb fragment: ac_1F and ac_169R
B2(aa33-1219)^{S876F}	pAC148	Reintroduction of the edge-π interaction (S>F) to B2	HA	Ligation of two fragments of 1.1kb and 2.9kb from pAC141 with 5.3kb fragment of pSA25 (EcoRI/XhoI)	1.1kb fragment: ac_172F and ac_21R;<br>2.9kb fragment: ac_1F and ac_173R
B2(528-921)	B2-HG	Expression of HormR and GAIN domains for crystallization	HA/TST	Ligation of 1.2kb insert from hB2 into pHLsec backbone (4.7kb)	B2_528-X_SacI<br>B2_y-921_KpnI

Vectors used for the expression of the ectodomain constructs

Receptor construct	Descriptions	Tags	Construction method	Primers used
pTwist hB2^{wt}	human ADGRB2 wild type construct in vector pTwist	Twin-Strep + Fc	Synthesized by Twist Bioscience	-
pTwist hB2^{basic}	hB2 with mutations S876F/D890S/E892Q/N893G	Twin-Strep + Fc	Synthesized by Twist Bioscience	-
pTwist hB2^{F1}	hB2 with introduced edge-π interaction and flap1 exchange from ADGRL1	Twin-Strep + Fc	Synthesized by Twist Bioscience	-
pTwist hB2^{F2}	hB2 with introduced edge-π interaction and flap2 exchange from ADGRL1	Twin-Strep + Fc	Synthesized by Twist Bioscience	-

pTwist hB2^{F1+2}	hB2 with introduced edge-π interaction and flap1+2 exchange from ADGRL1	Twin-Strep + Fc	Synthesized by Twist Bioscience	-
pTwist hB2^{F1BC}	hB2 with introduced edge-π interaction and flap1+1B + 1C exchange from ADGRL1	Twin-Strep + Fc	Synthesized by Twist Bioscience	-
pTwist hB2^{F1BC+2}	hB2 with introduced edge-π interaction and flap1+1B + 1C +2 exchange from ADGRL1	Twin-Strep + Fc	Synthesized by Twist Bioscience	-
pTwist rL1^{F1}	rat ADGRL1 wild type construct with flap1 exchange from ADGRB2	Twin-Strep + Fc	Synthesized by Twist Bioscience	-
pTwist rL1^{F2}	rat ADGRL1 wild type construct with flap2 exchange from ADGRB2	Twin-Strep + Fc	Synthesized by Twist Bioscience	-
pTwist rL1^{F1+2}	rat ADGRL1 wild type construct with flap1+2 exchange from ADGRB2	Twin-Strep + Fc	Synthesized by Twist Bioscience	-
pHLsec rL1^{wt}	rat ADGRL1 wild type construct in vector pHLsec	HA + Fc-His	Ligation with AgeI and KpnI	Amplification: rL1_HG_AgeI_for/ rL1_HG_AgeI_rev
pHLsec rL1^{F803W}	rL1 with F803W mutation	HA + Fc-His	Mutagenesis on vector pMA, Ligation with AgeI and KpnI	Mutagenesis: F803W_for/ F803W_rev Amplification: rL1_HG_AgeI_for/ rL1_HG_AgeI_rev
pHLsec rL1^{F803Y}	rL1 with F803Y mutation	HA + Fc-His	Mutagenesis on vector pMA, Ligation with AgeI and KpnI	Mutagenesis: F803Y_for F803Y_rev Amplification: rL1_HG_AgeI_for/ rL1_HG_AgeI_rev
pHLsec rL1^{F803A}	rL1 with F803A mutation	HA + Fc-His	Mutagenesis on vector pMA, Ligation with AgeI and KpnI	Mutagenesis: F803A_for F803A_rev Amplification: rL1_HG_AgeI_for/ rL1_HG_AgeI_rev

pHLsec rL1^{F803L}	rL1 with F803L mutation	HA + Fc-His	Mutagenesis on vector pMA, Ligation with AgeI and KpnI	Mutagenesis: F803L_for/ F803L_rev Amplification: rL1_HG_AgeI_for/ rL1_HG_AgeI_rev
pHLsec rL1^{H836S}	rL1 with H836S mutation	HA + Fc-His	Mutagenesis on vector pMA, Ligation with AgeI and KpnI	Mutagenesis: H836S_for/ H836S_rev Amplification: rL1_HG_AgeI_for/ rL1_HG_AgeI_rev
pHLsec hB3^{wt}	human ADGRB3 wild type construct in vector pHLsec	HA + Fc-His	Ligation with AgeI and KpnI	Amplification: rL1_HG_AgeI_for/ rL1_HG_AgeI_rev
pHLsec hB3^{R855H}	hB3 R855H variant	HA + Fc-His	Mutagenesis on vector pMA, Ligation with AgeI and KpnI	Mutagenesis: R855H_for/R855H_rev Amplification: hB3_HG_AgeI_for / hB3_HG_KpnI_rev
pHLsec hB3$^{L821W/R855H}$	hB3 L821W/R855H	HA + Fc-His	Mutagenesis on vector pMA, Ligation with AgeI and KpnI	Mutagenesis: R855H_for/R855H_rev/ L821W_for/L821W_rev Amplification: hB3_HG_AgeI_for / hB3_HG_KpnI_rev
pHLsec hB3$^{L821Y/R855H}$	hB3 L821Y/R855H	HA + Fc-His	Mutagenesis on vector pMA, Ligation with AgeI and KpnI	Mutagenesis: R855H_for/R855H_rev/ L821Y_for/L821Y_rev Amplification: hB3_HG_AgeI_for / hB3_HG_KpnI_rev
pHLsec hB3$^{L821F/R855H}$	hB3 mutant with L821F/R855H	HA + Fc-His	Mutagenesis on vector pMA, Ligation with AgeI and KpnI	Mutagenesis: R855H_for/R855H_rev/ L821F_for/L821F_rev Amplification: hB3_HG_AgeI_for / hB3_HG_KpnI_rev
pHLsec hB3$^{L821A/R855H}$	hB3 mutant with L821A/R855H	HA + Fc-His	Mutagenesis on vector pMA, Ligation with AgeI and KpnI	Mutagenesis: R855H_for/R855H_rev/ L821A_for/L821A_rev Amplification: hB3_HG_AgeI_for / hB3_HG_KpnI_rev
