# Supplementary material for: Structural basis of GAIN domain autoproteolysis and cleavage-resistance in the adhesion G-protein coupled receptors
Source: Nat Commun. 2026 Apr 6;17:3259. doi: 10.1038/s41467-026-71225-1 (PMC13062082; doi:10.1038/s41467-026-71225-1)
Supplement: Supplementary file 4 — Reporting Summary [file 41467_2026_71225_MOESM4_ESM.pdf]

nature portfolio

Corresponding author(s): Tobias Langenhan, Peter W. Hildebrand, Norbert Sträter

Last updated by author(s): 24.09.2025

Reporting Summary

Nature Portfolio wishes to improve the reproducibility of the work that we publish. This form provides structure for consistency and transparency in reporting. For further information on Nature Portfolio policies, see our Editorial Policies and the Editorial Policy Checklist.

Statistics

For all statistical analyses, confirm that the following items are present in the figure legend, table legend, main text, or Methods section.

n/a	Confirmed	
☐	☒	The exact sample size (*n*) for each experimental group/condition, given as a discrete number and unit of measurement
☐	☒	A statement on whether measurements were taken from distinct samples or whether the same sample was measured repeatedly
☐	☒	The statistical test(s) used AND whether they are one- or two-sided<br>*Only common tests should be described solely by name; describe more complex techniques in the Methods section.*
☒	☐	A description of all covariates tested
☐	☒	A description of any assumptions or corrections, such as tests of normality and adjustment for multiple comparisons
☐	☒	A full description of the statistical parameters including central tendency (e.g. means) or other basic estimates (e.g. regression coefficient) AND variation (e.g. standard deviation) or associated estimates of uncertainty (e.g. confidence intervals)
☐	☒	For null hypothesis testing, the test statistic (e.g. *F*, *t*, *r*) with confidence intervals, effect sizes, degrees of freedom and *P* value noted<br>*Give P values as exact values whenever suitable.*
☒	☐	For Bayesian analysis, information on the choice of priors and Markov chain Monte Carlo settings
☒	☐	For hierarchical and complex designs, identification of the appropriate level for tests and full reporting of outcomes
☒	☐	Estimates of effect sizes (e.g. Cohen's *d*, Pearson's *r*), indicating how they were calculated

Our web collection on statistics for biologists contains articles on many of the points above.

Software and code

Policy information about availability of computer code

Data collection	QM/MM : CHARMM46, / TURBOMOLE 7.4 with a custom patch of the CHARMM/TURBOMOLE interface. For MD: GROMACS/2023.1, NAMD2
Data analysis	Python 3.9: matplotlib, scipy, scikit-learn, mdciao, mdtraj. Visualization: VMD1.9.3, PyMOL3, GelAnalyzer version 23.1.1, GraphPadPrism 10.4.1, XDS (BUILT 20210323), AIMLESS 0.7.7, STARANISO 2.3.74, PHENIX 1.18.2_3874

For manuscripts utilizing custom algorithms or software that are central to the research but not yet described in published literature, software must be made available to editors and reviewers. We strongly encourage code deposition in a community repository (e.g. GitHub). See the Nature Portfolio guidelines for submitting code & software for further information.

Data

Policy information about availability of data

All manuscripts must include a data availability statement. This statement should provide the following information, where applicable:

- Accession codes, unique identifiers, or web links for publicly available datasets
- A description of any restrictions on data availability
- For clinical datasets or third party data, please ensure that the statement adheres to our policy

Data of the MD systems for the uncleaved GAIN domain model, ester intermediate and cleaved GAIN domain model, MD trajectories, used models, minimized QM/MM conformations and example input files for running QM/MM as well as a list of QM atoms will be available in our provided ZENODO repository, which will be release upon publication. The crystal structure of the ADGRB2 HormR-GAIN domain structure has been deposited in the protein data bank under pdb id 8oek.

Should any raw data files be needed in another format they are available from the corresponding author upon reasonable request. The images of gels and western blots have been deposited with their analysis at DOI:10.5281/zenodo.1549016.

Research involving human participants, their data, or biological material

Policy information about studies with human participants or human data. See also policy information about sex, gender (identity/presentation), and sexual orientation and race, ethnicity and racism.

Reporting on sex and gender	*Use the terms sex (biological attribute) and gender (shaped by social and cultural circumstances) carefully in order to avoid confusing both terms. Indicate if findings apply to only one sex or gender; describe whether sex and gender were considered in study design; whether sex and/or gender was determined based on self-reporting or assigned and methods used.<br>Provide in the source data disaggregated sex and gender data, where this information has been collected, and if consent has been obtained for sharing of individual-level data; provide overall numbers in this Reporting Summary. Please state if this information has not been collected.<br>Report sex- and gender-based analyses where performed, justify reasons for lack of sex- and gender-based analysis.*
Reporting on race, ethnicity, or other socially relevant groupings	*Please specify the socially constructed or socially relevant categorization variable(s) used in your manuscript and explain why they were used. Please note that such variables should not be used as proxies for other socially constructed/relevant variables (for example, race or ethnicity should not be used as a proxy for socioeconomic status).<br>Provide clear definitions of the relevant terms used, how they were provided (by the participants/respondents, the researchers, or third parties), and the method(s) used to classify people into the different categories (e.g. self-report, census or administrative data, social media data, etc.)<br>Please provide details about how you controlled for confounding variables in your analyses.*
Population characteristics	*Describe the covariate-relevant population characteristics of the human research participants (e.g. age, genotypic information, past and current diagnosis and treatment categories). If you filled out the behavioural & social sciences study design questions and have nothing to add here, write "See above."*
Recruitment	*Describe how participants were recruited. Outline any potential self-selection bias or other biases that may be present and how these are likely to impact results.*
Ethics oversight	*Identify the organization(s) that approved the study protocol.*

Note that full information on the approval of the study protocol must also be provided in the manuscript.

Field-specific reporting

Please select the one below that is the best fit for your research. If you are not sure, read the appropriate sections before making your selection.

☒ Life sciences ☐ Behavioural & social sciences ☐ Ecological, evolutionary & environmental sciences

For a reference copy of the document with all sections, see nature.com/documents/nr-reporting-summary-flat.pdf

Life sciences study design

All studies must disclose on these points even when the disclosure is negative.

Sample size	No sample size calculation was performed.
Data exclusions	For the analysis of the cleavage fraction of the full-length receptors (Figure 6CF) no data was excluded. For the analysis of the cleavage fraction of the ectodomain constructs (Figure 7C) and for the analysis of the cleavage kinetics of the ectodomain constructs (Figure 7E and Figure S15) data points were excluded if the background of the western blot did not allow for a quantification due to artefacts or if the total intensity of cleaved and uncleaved protein bands was too low for quantification as a result of protein decay during the 14 day experiment. Thle latter was the case for experiments hB2 F1 low and hB2 F1BC+2 (Figure S15).
Replication	The number of replicates for the analysis of the cleavage fraction of the full length receptor constructs (Figure 6CF) is indicated by the number of data points in the plot. The number of replicates in the analysis of the cleavage fraction of the ectodomain constructs (Figure 7C) is indicated by the number of data points in the plot. The number of replicates in the cleavage kinetics experiments (Figure 7E and Figure S15) is specified in the legend of Figure S15.
Randomization	Allocation of cells for specific experiments was random and all cells were maintained under identical culture conditions. Covariates were not relevant in this study as experimental and control experiments were performed in parallel.
Blinding	Blinding was not required for the analysis of cleavage fraction via western blotting due to their quantitative nature. Blinding was not possible for solving the crystal structure, since the phase problem was solved via molecular replacement, requiring prior knowledge of the target protein and expected structure.

Behavioural & social sciences study design

All studies must disclose on these points even when the disclosure is negative.

Study description	*Briefly describe the study type including whether data are quantitative, qualitative, or mixed-methods (e.g. qualitative cross-sectional, quantitative experimental, mixed-methods case study).*
Research sample	*State the research sample (e.g. Harvard university undergraduates, villagers in rural India) and provide relevant demographic information (e.g. age, sex) and indicate whether the sample is representative. Provide a rationale for the study sample chosen. For studies involving existing datasets, please describe the dataset and source.*
Sampling strategy	*Describe the sampling procedure (e.g. random, snowball, stratified, convenience). Describe the statistical methods that were used to predetermine sample size OR if no sample-size calculation was performed, describe how sample sizes were chosen and provide a rationale for why these sample sizes are sufficient. For qualitative data, please indicate whether data saturation was considered, and what criteria were used to decide that no further sampling was needed.*
Data collection	*Provide details about the data collection procedure, including the instruments or devices used to record the data (e.g. pen and paper, computer, eye tracker, video or audio equipment) whether anyone was present besides the participant(s) and the researcher, and whether the researcher was blind to experimental condition and/or the study hypothesis during data collection.*
Timing	*Indicate the start and stop dates of data collection. If there is a gap between collection periods, state the dates for each sample cohort.*
Data exclusions	*If no data were excluded from the analyses, state so OR if data were excluded, provide the exact number of exclusions and the rationale behind them, indicating whether exclusion criteria were pre-established.*
Non-participation	*State how many participants dropped out/declined participation and the reason(s) given OR provide response rate OR state that no participants dropped out/declined participation.*
Randomization	*If participants were not allocated into experimental groups, state so OR describe how participants were allocated to groups, and if allocation was not random, describe how covariates were controlled.*

Ecological, evolutionary & environmental sciences study design

All studies must disclose on these points even when the disclosure is negative.

Study description	*Briefly describe the study. For quantitative data include treatment factors and interactions, design structure (e.g. factorial, nested, hierarchical), nature and number of experimental units and replicates.*
Research sample	*Describe the research sample (e.g. a group of tagged Passer domesticus, all Stenocereus thurberi within Organ Pipe Cactus National Monument), and provide a rationale for the sample choice. When relevant, describe the organism taxa, source, sex, age range and any manipulations. State what population the sample is meant to represent when applicable. For studies involving existing datasets, describe the data and its source.*
Sampling strategy	*Note the sampling procedure. Describe the statistical methods that were used to predetermine sample size OR if no sample-size calculation was performed, describe how sample sizes were chosen and provide a rationale for why these sample sizes are sufficient.*
Data collection	*Describe the data collection procedure, including who recorded the data and how.*
Timing and spatial scale	*Indicate the start and stop dates of data collection, noting the frequency and periodicity of sampling and providing a rationale for these choices. If there is a gap between collection periods, state the dates for each sample cohort. Specify the spatial scale from which the data are taken*
Data exclusions	*If no data were excluded from the analyses, state so OR if data were excluded, describe the exclusions and the rationale behind them, indicating whether exclusion criteria were pre-established.*
Reproducibility	*Describe the measures taken to verify the reproducibility of experimental findings. For each experiment, note whether any attempts to repeat the experiment failed OR state that all attempts to repeat the experiment were successful.*
Randomization	*Describe how samples/organisms/participants were allocated into groups. If allocation was not random, describe how covariates were controlled. If this is not relevant to your study, explain why.*
Blinding	*Describe the extent of blinding used during data acquisition and analysis. If blinding was not possible, describe why OR explain why blinding was not relevant to your study.*

Did the study involve field work? ☐ Yes ☐ No

Field work, collection and transport

Field conditions	*Describe the study conditions for field work, providing relevant parameters (e.g. temperature, rainfall).*
Location	*State the location of the sampling or experiment, providing relevant parameters (e.g. latitude and longitude, elevation, water depth).*
Access & import/export	*Describe the efforts you have made to access habitats and to collect and import/export your samples in a responsible manner and in compliance with local, national and international laws, noting any permits that were obtained (give the name of the issuing authority, the date of issue, and any identifying information).*
Disturbance	*Describe any disturbance caused by the study and how it was minimized.*

Reporting for specific materials, systems and methods

We require information from authors about some types of materials, experimental systems and methods used in many studies. Here, indicate whether each material, system or method listed is relevant to your study. If you are not sure if a list item applies to your research, read the appropriate section before selecting a response.

Materials & experimental systems

n/a	Involved in the study	
☐	☒	Antibodies
☐	☒	Eukaryotic cell lines
☒	☐	Palaeontology and archaeology
☒	☐	Animals and other organisms
☒	☐	Clinical data
☒	☐	Dual use research of concern
☒	☐	Plants

Methods

n/a	Involved in the study	
☒	☐	ChIP-seq
☒	☐	Flow cytometry
☒	☐	MRI-based neuroimaging

Antibodies

Antibodies used

Ectodomain purification and autoproteolysis assays
- Strep-Tactin HRP conjugate (Life Sciences; 2-1502-001)
- HA-antibody HRP (Miltenyi Biotech; 130-091-972)
- Anti-His6 peroxidase (Sigma-Aldrich; 11965085001

Full-length autoproteolysis assays
- rabbit α-HA (Cell Signaling Technology, #C29F4; RRID:AB_1549585)
- mouse α-tubulinβ (DSHB e7, RRID: AB_528499)
- IRDye(R) 680RD Goat anti-Rabbit IgG secondary antibody (#926-68071) (Licor)
- IRDye(R) 800CW Goat anti-Mouse IgG secondary antibody (#926-32210) (Licor)

Antibodies were diluted according to the supplier's manual/instructions

Validation

Antibodies were used in the experiments to quantify the fraction of cleaved and uncleaved protein using fused tags. For the ectodomain constructs, the absence of bands of cleaved protein for cleavage deficient variants and the absence of bands of uncleaved protein for variants showing full cleavage activity and the empty vector experiments validate the antibodies used. For the full-length constructs, empty-vector-transfected cells were included in all experiments to ensure the specificities of the primary antibodies and α-tubulinβ for the detection of tubulinβ was used as a loading control for western blotting.

Eukaryotic cell lines

Policy information about cell lines and Sex and Gender in Research

Cell line source(s)	HEK293T, German Collection of Microorganisms and Cell Culture (Braunschweig, Germany), #ACC635<br>HEK293S GnTI-, Dr. Yvonne Jones, Oxford University
Authentication	Cell lines were maintained and authenticated by the supplier, no additional authentication was performed by the authors of this study.
Mycoplasma contamination	Cell lines were regularly tested for mycoplasma contamination (approximately once every 3 months) by the authors and tested negative.
Commonly misidentified lines<br>(See ICLAC register)	No commonly misidentified cell line was used in this study.

Plants

Seed stocks	*Report on the source of all seed stocks or other plant material used. If applicable, state the seed stock centre and catalogue number. If plant specimens were collected from the field, describe the collection location, date and sampling procedures.*
Novel plant genotypes	*Describe the methods by which all novel plant genotypes were produced. This includes those generated by transgenic approaches, gene editing, chemical/radiation-based mutagenesis and hybridization. For transgenic lines, describe the transformation method, the number of independent lines analyzed and the generation upon which experiments were performed. For gene-edited lines, describe the editor used, the endogenous sequence targeted for editing, the targeting guide RNA sequence (if applicable) and how the editor was applied.*
Authentication	*Describe any authentication procedures for each seed stock used or novel genotype generated. Describe any experiments used to assess the effect of a mutation and, where applicable, how potential secondary effects (e.g. second site T-DNA insertions, mosiacism, off-target gene editing) were examined.*
